# From Brain to Heart: Possible Role of Amyloid-β in Ischemic Heart Disease and Ischemia-Reperfusion Injury

**DOI:** 10.3390/ijms21249655

**Published:** 2020-12-17

**Authors:** Giulia Gagno, Federico Ferro, Alessandra Lucia Fluca, Milijana Janjusevic, Maddalena Rossi, Gianfranco Sinagra, Antonio Paolo Beltrami, Rita Moretti, Aneta Aleksova

**Affiliations:** 1 Cardiothoracovascular Department, Azienda Sanitaria Universitaria Giuliano Isontina (ASUGI) and University of Trieste, 34100 Trieste, Italy; gagnogiulia@gmail.com (G.G.); fferro@units.it (F.F.); alessandrafluca@live.it (A.L.F.); mjanjusevic@units.it (M.J.); maddalenarossi93@gmail.com (M.R.); gianfranco.sinagra@asugi.sanita.fvg.it (G.S.); 2 Department of Medicine (DAME), University of Udine, 33100 Udine, Italy; antonio.beltrami@uniud.it; 3 Department of Internal Medicine and Neurology, Neurological Clinic, 34100 Trieste, Italy; moretti@units.it

**Keywords:** amyloid beta, Aβ1-40, ischemic heart disease, myocardial infarction, atherosclerosis, Alzheimer’s disease, cerebral amyloid angiopathy, ischemia-reperfusion injury, BACE1, cardiovascular mortality

## Abstract

Ischemic heart disease (IHD) is among the leading causes of death in developed countries. Its pathological origin is traced back to coronary atherosclerosis, a lipid-driven immuno-inflammatory disease of the arteries that leads to multifocal plaque development. The primary clinical manifestation of IHD is acute myocardial infarction (AMI),) whose prognosis is ameliorated with optimal timing of revascularization. Paradoxically, myocardium re-perfusion can be detrimental because of ischemia-reperfusion injury (IRI), an oxidative-driven process that damages other organs. Amyloid-β (Aβ) plays a physiological role in the central nervous system (CNS). Alterations in its synthesis, concentration and clearance have been connected to several pathologies, such as Alzheimer’s disease (AD) and cerebral amyloid angiopathy (CAA). Aβ has been suggested to play a role in the pathogenesis of IHD and cerebral IRI. The purpose of this review is to summarize what is known about the pathological role of Aβ in the CNS; starting from this evidence, we will illustrate the role played by Aβ in the development of coronary atherosclerosis and its possible implications in the pathophysiology of IHD and myocardial IRI. Better elucidation of Aβ’s contribution to the molecular pathways underlying IHD and IRI could be of great help in developing new therapeutic strategies.

## 1. Introduction

Since 2000, the World Health Organization (WHO) has identified ischemic heart disease (IHD) as the most prevalent cause of death worldwide [1]. In Italy, despite a decrease in IHD-related mortality observed from 2004 to 2014, IHD remains one of the leading causes of death [2].

IHD is defined as a condition characterized by the evidence of myocardial necrosis in a clinical setting consistent with acute myocardial ischemia [3]. In most cases, the pathological basis of IHD is coronary atherosclerosis, a systemic, lipid-driven immune-inflammatory disease of medium-sized and large arteries leading to multifocal plaque development [4]. Coronary atherosclerosis has been a field of intense investigation, and although many risk factors for acute coronary syndrome (ACS) development have been pointed out [5], identifying healthy individuals who are at high risk for ACS remains a challenge and hampers the development of targeted prevention strategies [4]. Amyloid-β (Aβ) has been recently proposed to be involved in the pathogenesis of coronary atherosclerosis [6]. The implication of Aβ in the pathophysiology of myocardial ischemia could be of great help in identifying individuals at higher risk of developing acute myocardial infarction (AMI) with a poor prognosis, eventually leading to the development of new strategies to reduce the risk of IHD and to improve the prognosis of infarcted patients. Aβ peptides are mostly known for their pathogenic role in the central nervous system (CNS), leading to Alzheimer’s disease (AD). Interestingly, new evidence of Aβ involvement in cardiovascular diseases has led to the development of a brain–heart crosstalk hypothesis. Despite it being known that AD and IHD share various risk factors, such as hypertension, diabetes, dyslipidemia, obesity and cigarette smoking, the exact prevalence of IHD among patients with AD has not been described, and evidence of a possible common pathogenesis of these two conditions is still a matter of study [7]. 

The sustained or permanent occlusion of a coronary artery determines ischemia of the myocardium supplied by the narrowed vessel [8]. It is accepted that the best therapeutic strategy for AMI is early reperfusion [9]. However, regardless of the optimal timing of revascularization, the myocardium can suffer an oxidative-driven process called ischemia-reperfusion injury (IRI). Several emerging therapeutic strategies for preventing IRI, such as intermittent reperfusion (IPost), remote ischemic post-conditioning and hyperoxemia-hypothermia, have shown promising experimental results. However, it is not easy to translate them into daily clinical practice [8].

In the CNS, it has been shown that Aβ plays a synergistic role with cerebral ischemia-reperfusion in exacerbating neuronal damage by inducing glycogen synthase kinase 3β and protein phosphatase 2A activity, resulting in the phosphorylation of τ protein [10]. However, the role exerted by Aβ in exacerbating cardiac IRI still remains to be elucidated. 

The purpose of this review is to summarize what is known about the pathological role of Aβ, specifically focusing on its possible implication in IHD and IRI.

## 2. The Pathogenic Role of Aβ1-40 in the CNS

**Figure 1:**
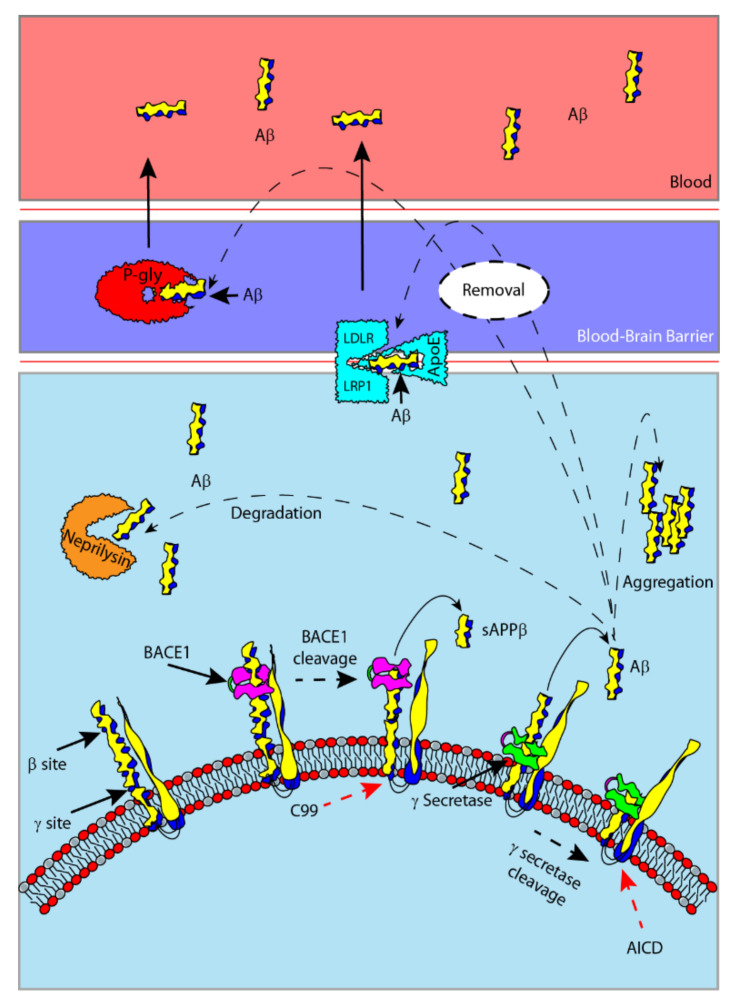
Schematic representation of amyloid-β (Aβ) generation from amyloid precursor protein (APP) processing by secretases on cell membranes. β-secretase cuts APP on the β-site, producing a soluble fragment (sAPPβ); while γ-secretase cuts on the γ-site, generating an intracellular fragment (AICD) and Aβ that is released. Aβ is degraded in the perivascular space by neprilysin, or inside endothelial cells and neurons. The transporters low-density lipoprotein receptor (LDLR) and LDLR-related protein 1 (LRP1) (after Aβ–apolipoprotein E (ApoE) complex binding) and P-glycoprotein allow Aβ to cross the blood–brain barrier to the blood, contributing to the increase of its plasmatic concentration. The impairment of Aβ clearance causes its aggregation in the perivascular space.

Aβs are peptides generated by the sequential proteolytic cleavage of the amyloid precursor protein (APP) through the activity of β- and γ-secretase (Figure 1). In particular, Aβ1-40 and Aβ1-42 are the most relevant from a pathophysiological standpoint [11]. Aβ plays a physiological role in the CNS [12] and alterations in its synthesis, processing and clearance can lead to different pathologies. Usually, Aβ is removed from the CNS by enzymatic degradation (e.g., neprilysin) via the circulatory system or the glymphatic pathway [13,14]. Aβ crosses the blood–brain barrier (BBB) and enters blood vessels through specific transporters. Apolipoprotein E (ApoE) binds Aβ with different affinity depending on isoforms and allows the passage of Aβ in the blood through mechanisms mediated by low-density lipoprotein receptor (LDLR) and LDLR-related protein 1 (LRP1) [15]. Aβ can also be transported through the BBB as a free monomer via P-glycoprotein [16] (Figure 1).

The first evidence that Aβ could be linked to pathological conditions emerged in Alzheimer’s disease, where the peptide is known to cause neurotoxicity. When Aβ is released in the perivascular space, it accumulates, causing senile plaques (SPs). Although large insoluble aggregates such as SPs do not cause memory impairment, Aβ has been discovered to have a neurotoxic effect when it forms oligomers [17]. Among the various forms of Aβ peptides, the tendency of Aβ1-42 to aggregate is five times higher than Aβ1-40, even though they differ in just two amino acids [18]. Aβ1-42 shows the highest tendency to aggregate due to its β-sheet conformation, and it is the main constituent of SPs, in association with the hyper-phosphorylated τ protein [19]. Aβ1-42 and Aβ1-40 interact during SP formation in a vicious circle in which Aβ1-42 promotes Aβ1-40 aggregation and vice versa, thus explaining their co-presence in SPs [20].

Recent controversial studies on AD patients have shown that Aβ peptides can also accumulate within neurons in different subcellular compartments, such as the endoplasmic reticulum, Golgi apparatus and endosomes [21]. Consistent with this notion, the enzymatic activity of transmembrane proteins β-secretase (BACE1) and γ-secretase is enhanced when their active site lays within acid organelles (i.e., endoplasmic reticulum, Golgi apparatus and endosomes) [22,23,24]. Furthermore, many data suggest that the intracellular accumulation of Aβ also plays a pivotal role in the development of AD [21], with intracellular aggregates associated with cytotoxicity, apoptosis and neuronal cell death [21,25].

It has been observed that around 62–92% of AD patients develop cerebral amyloid angiopathy (CAA) [26]. CAA results from the deposition of Aβ in the cerebral leptomeningeal and parenchymal arteries and in the arteriolar walls. While white matter lesions and infarcts do not seem to influence amyloid pathology, some other pieces of evidence suggest that increased vascular risk is related to increased amyloid burden [27]. Furthermore, while vascular brain injuries and amyloid have an additive and independent impact on brain integrity, vascular risk factors might potentiate amyloid impact on the cortical thickness in brain regions vulnerable to AD [28,29].

While Aβ1-42 plays a crucial role in SP formation, Aβ1-40 seems to be the most responsible peptide in CAA. This difference may reflect a diverse Aβ subtype source, with Aβ1-42 deriving from neurons and astrocytes and Aβ1-40 having a vascular origin [30]. In vessels affected by CAA, local muscle and elastic elements are lost and replaced by amyloid fibrils, thereby weakening the vessel’s overall structure. Consequently, CAA predisposes to cerebral infarction and cerebral hemorrhage. However, the clinical effects of CAA in AD are mostly silent or “masked” by the higher degree of neuronal dysfunction. As mentioned above, ApoE plays an important role in Aβ metabolism, which is supposed to be dysregulated in carriers of ApoE ε4 polymorphism, known to be at increased risk of developing AD. Interestingly, VEGF has been shown to play a neuroprotective role in ApoE ε4 mice. This effect may be due to VEGF-mediated angiogenesis, which prevents ischemia and downstream neurodegeneration [31].

Nonetheless, significant cerebral infarctions with focal neurological deficits can occur in some AD patients, and CAA is a significant cause of fatal intracerebral (lobar) hemorrhage. CAA may also contribute to white matter lesions (myelin loss) in AD by inducing ischemia through auto-regulatory dysfunction, which in turn creates an inflammatory status, inducing hyper-phosphorylation of τ proteins. These reactions cause neurofibrillary degeneration, with the consequent deposition of SPs. Although the Aβ protein deposited within blood vessels of patients with AD is similar in chemical composition to that deposited in the brain parenchyma in SPs, there is no clear relationship between CAA and AD. When CAA is high, SP formation may be lower and vice versa [29].

In subjects with CAA, Aβ increases oxidative stress, which causes endothelial cell apoptosis with consequent damage to the BBB [32]. It has also been seen that Aβ1-40 promotes inflammation in vitro [33] and in vivo [34], stimulating the production of pro-inflammatory cytokines, increasing the permeability of the endothelium and the expression of adhesion molecules. A small number of specific inherited forms of CAA with cerebral hemorrhage are associated with autosomal dominant mutations in APP and other genes (cystatin-C, transthyretin, gelsolin, ABrit and ADan). In most AD cases, CAA does not associate clearly with any genetic risk factor other than the APO E beta4 allele, which appears to increase the severity of CAA in a dose-dependent manner, especially within the occipital cortex. Genotype/phenotype correlations may explain CAA development in AD and other disorders [30].

In addition to its direct cytotoxicity, Aβ is also associated with micro-thrombotic events. Fibrillary aggregates of Aβ activate platelets through a mechanism similar to the one mediated by collagen and glycoprotein VI following endothelial injury. In particular, Aβ1-40 has a more pronounced ability to induce platelet aggregation than Aβ1-42 [35]. Aβ activation of platelets promotes micro-thrombotic events, reducing blood flow and causing cerebral ischemia, thereby leading to CAA [36,37]. In the APP23 transgenic mouse model, which is well-known for developing Aβ deposits in the brain parenchyma and cerebral vessels, platelets are persistently in a pre-activated state associated with an increased risk of thrombus formation. Of note, these mice were found to develop cerebrovascular and cardiac complications [38]. Other studies found that Aβ and APP have an essential role in the interaction between platelets and collagen in arteries. Therefore, APP is directly involved in arterial thrombus formation [39].

## 3. Pathogenic Role of Aβ1-40 in Atherosclerosis 

The production of APP and Aβ have been documented in many tissues other than the CNS, such as muscle, skin, adipose tissue, intestine [40], endothelium and heart [38,41], and it seems that the abundance of circulating Aβ could be linked to pathologies other than AD, such as atherosclerosis [6]. On the other hand, although APP expression is well-documented in endothelial cells and cardiomyocytes, its physiological and pathological role in the cardiovascular system is poorly understood [42]. Nonetheless, APP might participate in atheroma formation and thrombosis—two pivotal mechanisms in the pathophysiology of IHD.

Endothelial cells are sensitive to biochemical, inflammatory physical stimuli allowing for the autoregulation of blood vessels in the case of blood flow variations [43]. These elegant mechanisms can be perturbed by external factors, leading to the development of atherosclerosis [44]. In particular, blood flow variations induce hypoxia inducible factor-1α (HIF-1α), which increases endothelial cell permeability and promotes lipids accumulation within the tunica intima of vessels—a mechanism that might play a key role in the initiation of arteriosclerotic plaque formation [45]. Besides, oscillatory shear stress increases the activity of transcription factors such as HIF-1α, nuclear factor kappa-light-chain-enhancer of activated B cells (NF-κB), GATA-1, GATA-4 as well as cytokines (e.g., tumor necrosis factor- α, TNF-α), all of which regulate BACE1 transcription [46,47,48,49,50,51,52]. 

**Figure 2:**
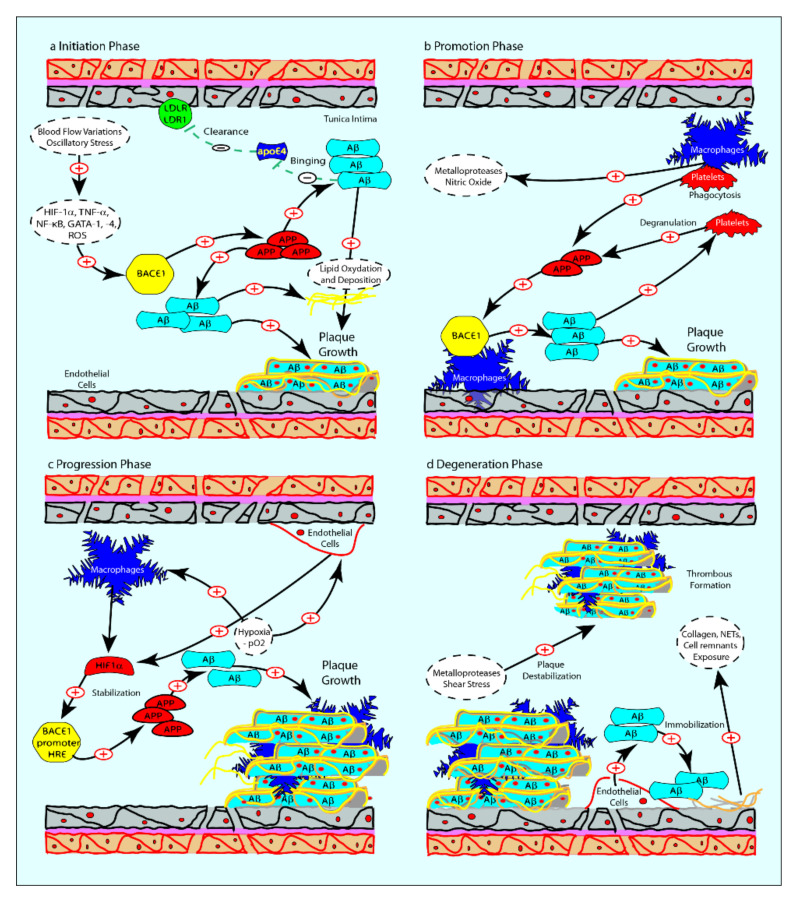
(**a**) Schematic representation of Aβ contribution to atherosclerotic plaque formation. Flow variations cause endothelial cell dysfunction and induce HIF-1α, TNF-α, NF-κB, GATA-1 and GATA-4, causing the increase of APP processing and Aβ production in endothelial cells. Aβ also promotes the activation of endothelial cells, which increase LDLR and LRP1 on the cell membrane. As a result, there is an accumulation of lipids in the tunica intima of blood vessels; (**b**) during the promotion phase, macrophages phagocytize APP, which is then processed by BACE1, increasing local and circulating Aβ concentration and stimulating the production of nitric oxide and metalloproteases. The presence of Aβ in atherosclerotic plaques is also explained by its production in both endothelial cells and platelets; (**c**) the hypoxic condition leads to HIF-1α binding HRE on the BACE1 promoter, in both endothelial cells and macrophages, increasing Aβ production and formation of the atheroma; (**d**) in the degeneration phase, matrix metalloproteases and apoptosis destabilize atherosclerotic plaque, causing cell death and the exposition of collagen, neutrophil extracellular traps (NETs) and membranes on which Aβ can be immobilized, causing extracellular amyloid deposits.

Atherosclerotic plaque formation can be described as a four-stage process: initiation, promotion, progression and degeneration. Aβ is implied not only in the initiation of plaque formation but also in other plaque development steps. It has been demonstrated that Aβ1-40 favors the deposition and oxidation of lipids in vessels, stimulating arteriosclerosis initiation [53]. ApoE and, in particular, its isoform ApoE4, which is implied in atherosclerotic plaque formation, has a lower affinity to Aβ than other isoforms, thus causing a reduction in Aβ clearance via LDLR and LRP1, thus promoting its accumulation in atherosclerotic plaques [54] (Figure 2A). Macrophages, which are pivotal in the so-called “promotion phase,” phagocytize APP, which is then processed by BACE1, increasing both local and circulating levels of Aβ as well as stimulating the production of nitric oxide and metalloproteases [55,56]. The presence of Aβ in atherosclerotic plaques is also enhanced by the hypoxic environment, which promotes the stabilization of HIF-1α, which in turn increases the processing of Aβ in endothelial cells and macrophages by binding the hypoxia-responsive element (HRE) on the BACE1 gene promoter [57]. As previously mentioned, Aβ favors platelet aggregation and degranulation, providing a further boost to plaque progression [10,36] (Figure 2B). The result is the formation of atheroma in coronary arteries that narrows the lumen and reduces the oxygen and blood supply to the downstream tissue, which becomes ischemic [57] (Figure 2C). Ischemia leads to endothelial cell death, plaque degeneration and rupture, thrombus formation and consequent collagen exposure. Moreover, ischemia promotes the formation of neutrophil extracellular traps (NETs). All these events can also lead to the release of intracellular Aβ1-40, which can be rapidly immobilized onto the exposed collagen and NETs, causing extracellular amyloid deposits [35,58,59] (Figure 2D).

## 4. Suggesting a Pathologic Role for Aβ1-40 in IHD

Recent studies have recognized the intracellular accumulation of Aβ1-40 in endothelial cells and cardiomyocytes as a direct cause of cytotoxicity [41], similar to what has been observed in neurons of patients affected by CAA [32,33,34]. Endothelial cells and cardiomyocytes exposed to Aβ1-40 manifest changes in their transcriptional profile, particularly of genes related to the ubiquitin-proteasome system, apoptosis, DNA damage and inflammation [41,47,60]. The molecular mechanisms underlying deregulation of BACE1 expression and activity, which leads to the accumulation of Aβ1-40 in cardiomyocytes and endothelial cells, are not yet understood [41]. It seems that all the established cardiovascular risk factors are associated with overexpression of BACE1 and over-production of Aβ1-40 that, in elderly individuals, is not counteracted by proteasome action due to the age-related lower efficiency of the system [47,48,49,61,62,63,64,65]. Moreover, the intracellular accumulation of Aβ1-40, particularly in the lumen of the endoplasmic reticulum (ER), worsens endothelial cell dysfunction, leading to the recruitment of monocytes and the accumulation of lipids.

**Figure 3:**
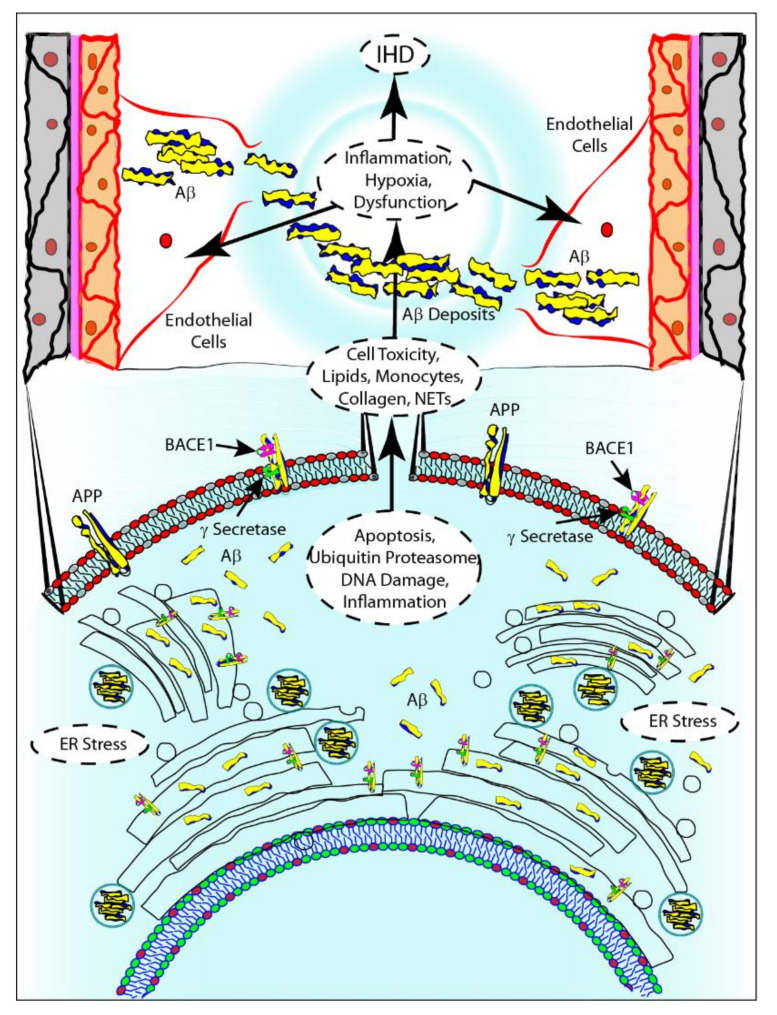
Aβ1-40 cytotoxicity and its possible involvement in the onset of atherosclerotic plaque. Aβ1-40 intracellular accumulation causes changes in the endothelial cells transcriptional profile, with a marked expression of genes related to ubiquitin proteasome system, apoptosis, DNA damage and inflammation. Cell death leads to the release of Aβ1-40, which can be rapidly immobilized onto the exposed collagen and NETs, causing extracellular amyloid deposits. Consequently, inflammation, endothelial damage and clot formation increase, leading to myocardial hypoxia, dysfunction and finally to the development of IHD, independent from coronary atherosclerosis.

As previously described, Aβ aggregates at the extracellular level in CAA. To date, there is no evidence of extracellular Aβ aggregates in coronary arteries; however, it cannot be excluded that this mechanism also contributes to the development of the disease. The intracellular Aβ1-40, released following endothelial cell death, could be rapidly immobilized onto the exposed collagen and NETs, causing extracellular amyloid deposits [58,59,66]. In turn, extracellular amyloid deposits could worsen inflammation and endothelial damage and increase clot formation, thus leading to myocardial hypoxia and dysfunction. In this scenario, the prolonged ischemic state could lead to the development of IHD independent of coronary atherosclerosis (Figure 3).

## 5. Suggesting a Pathologic Role for Aβ1-40 in IRI

Paradoxically, blood flow restoration after an ischemic event can worsen cellular dysfunction and a wide range of tissue damage; this condition is known as ischemia-reperfusion injury (IRI). IRI is a complex multifactorial process that may affect not only the ischemic organ, but also distant ones [67]. Because of the adverse effects of ischemia-reperfusion, it is of great interest to elucidate its molecular players: in this context, as described below, Aβ emerges as a new factor.

**Figure 4:**
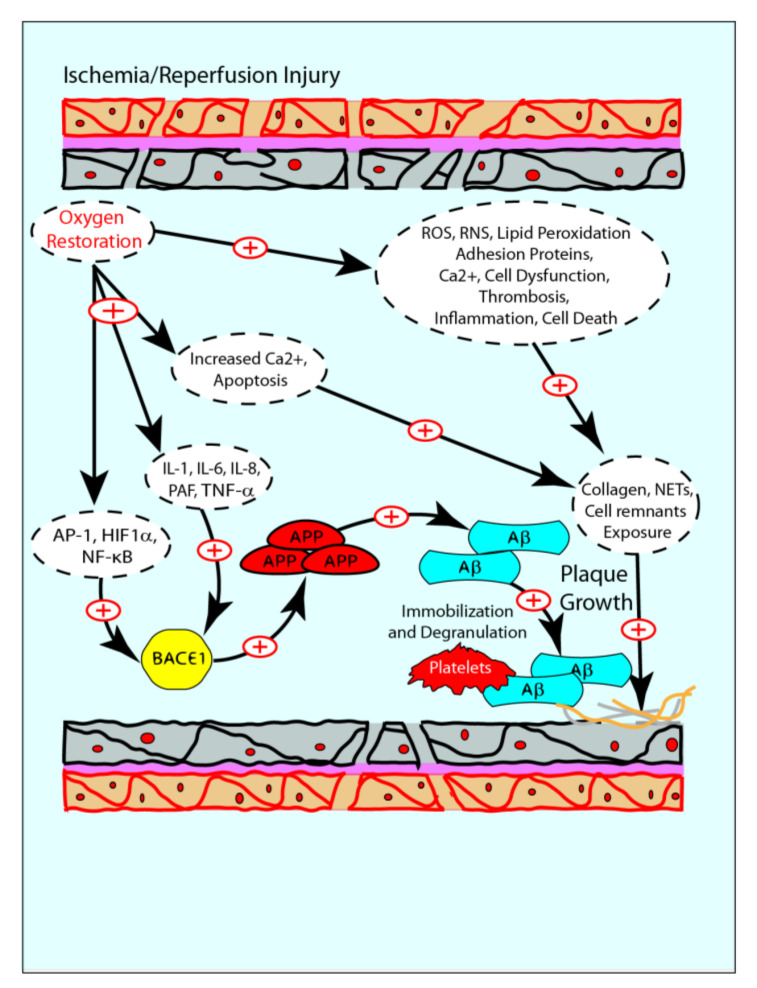
Schematic representation of Aβ–IRI relation. After reperfusion inflammation, pO_2_ changes and endothelial cell dysfunction create optimal conditions for Aβ to re-initiate the atherosclerotic plaques. Because of Aβ’s cytotoxic and pro-inflammatory effects, it could be hypothesized that it is involved in IRI as well.

Restoration of normal pO_2_ after reperfusion is associated with an increase in reactive oxygen species (ROS) and reactive nitrogen species (RNS), lipid peroxidation, enhanced expression of adhesion proteins, mitochondrial and intracytoplasmic Ca^2+^ accumulation, endothelial cell dysfunction, thrombosis, inflammation, reduction of nitric oxide (NO) and eventually cell death [68,69,70]. Moreover, reperfusion is associated with the increased production of some key transcription factors, including AP-1 [71], HIF-1α [72] and NF-κB [50]. All these transcription factors are related to APP processing and Aβ production [73], leading to an increase of Aβ1-40 concentration in the extra and intracellular spaces [74]. Furthermore, ischemia-reperfusion induces a systemic release of several cytokines (e.g., TNF-α, IL-1, IL-6, IL-8 and platelet-activating factor (PAF)) [75], causing the development of a systemic inflammatory state. Consequently, this inflammation favors an increase of APP expression and leads to increased Aβ production and a reduced Aβ uptake/degradation [76]. It has also been observed that, following reperfusion, the increased intracellular concentration of Ca^2+^ can stimulate the deposition of cytoplasmic Aβ, which alters cellular integrity and leads to apoptosis through the formation of nonspecific pores in the plasma membrane [77]. These data indicate that AMI and reperfusion are involved in inflammatory, apoptosis and oxidative processes [78,79,80] (Figure 4). 

Recent preclinical studies in CNS have confirmed the increased expression of proprotein convertase subtilisin/kexin type 9 (PCSK9) in endothelium after AMI and during acute myocardial reperfusion. It has also been demonstrated that PCSK9 expression is directly related to Aβ neural aggregation [81,82,83]. Some studies have also suggested that PCSK9 might increase Aβ deposition, degrading brain endothelial LRP1 with a consequent reduction in Aβ clearance [84,85]. In cardiac IRI, the effects of Aβ1-40 and roles of PCSK9 are still unknown; however, observations in the CNS might give fascinating insight to provide a better understanding IRI pathogenesis in the heart.

In the myocardium, after reperfusion of ischemic areas, the exposition of adhesion proteins, collagen, glycoprotein VI, NETs, ApoE and cell membranes is detectable because of cell death. The consequent release of intracellular Aβ1-40 leads to an increase in its plasmatic levels. As a result, amyloid deposits can form extracellularly, where they can activate and enhance platelet aggregation and degranulation, stimulating the formation of new atherosclerotic plaques or causing direct damage because of cytotoxicity [8,58,59,66,86] (Figure 4).

## 6. Conclusions and Future Directions

In addition to well-documented evidence of Aβ correlations with CNS pathologies, there is a growing amount of data on an association of Aβ with the pathophysiology of IHD, AMI and IRI. Although not yet clinically validated, recent data suggest that higher plasmatic levels of Aβ1-40 could be considered a predictor for the development of IHD and a marker of poorer outcomes. On this basis, Aβ1-40 could emerge as a new strategy to reduce the risk of IHD and improve its prognosis after AMI. Furthermore, balancing APP/Aβ synthesis, clearance and aggregation or reducing its cytotoxic and pro-inflammatory effects could have protective effects not only on the brain but also on the heart and vessels. In this view, Aβ1-40 could be considered a target for therapeutic approaches. Many attempts have been made to reduce or control Aβ1-40 levels, including lifestyle modification, use of statins, antihypertensive drugs and antithrombotic agents. However, none of them have demonstrated a clear effect in reducing Aβ1-40 concentration [87]. 

In search of a better clinical translation, some pharmacologic agents have also been proposed that could be useful in reducing ischemia-reperfusion injury. The most promising are cyclosporin A and exenatide, which preserve mitochondrial function and modulate the reperfusion injury salvage kinase pro-survival pathway, respectively [8]. Recently, because of the emerging evidence of Aβ1-40 effects in cerebral IRI after AMI, PCSK9i, humanin and gastrodin have been proposed as potential molecules to reduce Aβ-related adverse effects [88,89]. Better elucidation of the molecular pathways underlying ischemia-reperfusion injury could be of great help in developing new therapeutic strategies. However, despite the considerable amount of preclinical research, a relatively small number of compounds have reached clinical trial testing.

In conclusion, besides the known pathogenic role of Aβ in CNS, there are several pieces of molecular evidence supporting the role of Aβ both in the development of IHD and in the clinical course after AMI. However, Aβ’s connection with cardiac IRI needs to be further investigated. More data is needed to better understand the connection between plasmatic Aβ levels and their pathological implication, in order to use that knowledge in clinical trials and in the development of novel therapeutic strategies.

## Abbreviations

ACS	Acute coronary syndrome
AD	Alzheimer’s disease
AMI	Acute myocardial infarct
ApoE	Apolipoprotein E
APP	Amyloid protein precursor
Aβ	Amyloid-β
BACE1	Beta-site amyloid precursor protein cleaving enzyme 1
BBB	Blood–brain barrier
CAA	Cerebral amyloid angiopathy
CNS	Central nervous system
ER	Endoplasmic reticulum
HIF-1α	Hypoxia induced factor—1α
HRE	Hypoxia-responsive element
IHD	Ischemic heart disease
IRI	Ischemia-reperfusion injury
LDLR	Low-density lipoprotein receptor
LRP1	LDLR-related protein 1
NETs	Neutrophil extracellular traps
NF-κB	Nuclear factor kappa-light-chain-enhancer of activated B cells
PAF	Platelet-activating factor
PCSK9	Proprotein convertase subtilisin/Kexin type 9
SPs	Senile plaques
TNF-α	Tumor necrosis factor- α
WHO	World Health Organization
sAPPβ	Soluble amyloid precursor protein β
AICD	Amyloid-precursor protein intracellular domain
P-gly	P-glycoprotein
GATA-1	GATA binding factor 1
GATA-4	GATA binding factor 4
ROS	Reactive oxygen species
pO_2_	Partial O_2_ pressure
ER Stress	Endoplasmic reticulum stress
RNS	Reactive nitric species
IL-1, IL-6, IL-8	Interleukin
PAF	Platelet-activating factor
AP-1	Activator protein

## References

[1] WHO (2013). The Top 10 Causes of Death: Fact Sheet N°310.

[2] ISTAT L’evoluzione Della Mortalità per Causa: Le Prime 25 Cause di Morte.

[3] Thygesen K., Alpert J.S., Jaffe A.S., Chaitman B.R., Bax J.J., Morrow D.A., White H.D., Executive Group on behalf of the Joint European Society of Cardiology, American College of Cardiology, American Heart Association (2018). Fourth Universal Definition of Myocardial Infarction (2018). Circulation.

[4] Falk E., Nakano M., Bentzon J.F., Finn A.V., Virmani R. (2013). Update on acute coronary syndromes: The pathologists’ view. Eur. Heart J..

[5] Libby P., Buring J.E., Badimon L., Hansson G.K., Deanfield J., Bittencourt M.S., Tokgozoglu L., Lewis E.F. (2019). Atherosclerosis. Nat. Rev. Dis. Primers.

[6] Stamatelopoulos K., Sibbing D., Rallidis L.S., Georgiopoulos G., Stakos D., Braun S., Gatsiou A., Sopova K., Kotakos C., Varounis C. (2015). Amyloid-beta (1-40) and the risk of death from cardiovascular causes in patients with coronary heart disease. J. Am. Coll. Cardiol..

[7] Troncone L., Luciani M., Coggins M., Wilker E.H., Ho C.Y., Codispoti K.E., Frosch M.P., Kayed R., Del Monte F. (2016). Abeta Amyloid Pathology Affects the Hearts of Patients With Alzheimer’s Disease: Mind the Heart. J. Am. Coll. Cardiol..

[8] Hausenloy D.J., Yellon D.M. (2013). Myocardial ischemia-reperfusion injury: A neglected therapeutic target. J. Clin. Investig..

[9] Ibanez B., James S., Agewall S., Antunes M.J., Bucciarelli-Ducci C., Bueno H., Caforio A.L.P., Crea F., Goudevenos J.A., Halvorsen S. (2017). 2017 ESC Guidelines for the management of acute myocardial infarction in patients presenting with ST-segment elevation. Rev. Esp. Cardiol..

[10] Song B., Ao Q., Niu Y., Shen Q., Zuo H., Zhang X., Gong Y. (2013). Amyloid beta-peptide worsens cognitive impairment following cerebral ischemia-reperfusion injury. Neural. Regen. Res..

[11] Coronel R., Bernabeu-Zornoza A., Palmer C., Muniz-Moreno M., Zambrano A., Cano E., Liste I. (2018). Role of Amyloid Precursor Protein (APP) and Its Derivatives in the Biology and Cell Fate Specification of Neural Stem Cells. Mol. Neurobiol..

[12] Dawkins E., Small D.H. (2014). Insights into the physiological function of the beta-amyloid precursor protein: Beyond Alzheimer’s disease. J. Neurochem..

[13] Tarasoff-Conway J.M., Carare R.O., Osorio R.S., Glodzik L., Butler T., Fieremans E., Axel L., Rusinek H., Nicholson C., Zlokovic B.V. (2015). Clearance systems in the brain-implications for Alzheimer disease. Nat. Rev. Neurol..

[14] Hendrikx D., Smits A., Lavanga M., De Wel O., Thewissen L., Jansen K., Caicedo A., Van Huffel S., Naulaers G. (2019). Measurement of Neurovascular Coupling in Neonates. Front. Physiol..

[15] Bachmeier C., Shackleton B., Ojo J., Paris D., Mullan M., Crawford F. (2014). Apolipoprotein E isoform-specific effects on lipoprotein receptor processing. Neuromol. Med..

[16] Chiu C., Miller M.C., Monahan R., Osgood D.P., Stopa E.G., Silverberg G.D. (2015). P-glycoprotein expression and amyloid accumulation in human aging and Alzheimer’s disease: Preliminary observations. Neurobiol. Aging.

[17] Sengupta U., Nilson A.N., Kayed R. (2016). The Role of Amyloid-beta Oligomers in Toxicity, Propagation, and Immunotherapy. EBioMedicine.

[18] Acharya S., Srivastava K.R., Nagarajan S., Lapidus L.J. (2016). Monomer Dynamics of Alzheimer Peptides and Kinetic Control of Early Aggregation in Alzheimer’s Disease. Chemphyschem.

[19] Barage S.H., Sonawane K.D. (2015). Amyloid cascade hypothesis: Pathogenesis and therapeutic strategies in Alzheimer’s disease. Neuropeptides.

[20] Tran J., Chang D., Hsu F., Wang H., Guo Z. (2017). Cross-seeding between Abeta40 and Abeta42 in Alzheimer’s disease. FEBS Lett..

[21] Takahashi R.H., Nagao T., Gouras G.K. (2017). Plaque formation and the intraneuronal accumulation of beta-amyloid in Alzheimer’s disease. Pathol. Int..

[22] Oikawa N., Walter J. (2019). Presenilins and gamma-Secretase in Membrane Proteostasis. Cells.

[23] Koelsch G. (2017). BACE1 Function and Inhibition: Implications of Intervention in the Amyloid Pathway of Alzheimer’s Disease Pathology. Molecules.

[24] Cole S.L., Vassar R. (2007). The Alzheimer’s disease beta-secretase enzyme, BACE1. Mol. Neurodegener..

[25] Umeda T., Tomiyama T., Sakama N., Tanaka S., Lambert M.P., Klein W.L., Mori H. (2011). Intraneuronal amyloid beta oligomers cause cell death via endoplasmic reticulum stress, endosomal/lysosomal leakage, and mitochondrial dysfunction in vivo. J. Neurosci. Res..

[26] Bailey T.L., Rivara C.B., Rocher A.B., Hof P.R. (2004). The nature and effects of cortical microvascular pathology in aging and Alzheimer’s disease. Neurol. Res..

[27] Villeneuve S., Jagust W.J. (2015). Imaging Vascular Disease and Amyloid in the Aging Brain: Implications for Treatment. J. Prev. Alzheimers Dis..

[28] Chao L.L., Decarli C., Kriger S., Truran D., Zhang Y., Laxamana J., Villeneuve S., Jagust W.J., Sanossian N., Mack W.J. (2013). Associations between white matter hyperintensities and beta amyloid on integrity of projection, association, and limbic fiber tracts measured with diffusion tensor MRI. PLoS ONE.

[29] De la Torre J.C. (2004). Is Alzheimer’s disease a neurodegenerative or a vascular disorder? Data, dogma, and dialectics. Lancet Neurol..

[30] Tian J., Shi J., Mann D.M. (2004). Cerebral amyloid angiopathy and dementia. Panminerva Med..

[31] Moore A.M., Mahoney E., Dumitrescu L., De Jager P.L., Koran M.E.I., Petyuk V.A., Robinson R.A., Ruderfer D.M., Cox N.J., Schneider J.A. (2020). APOE ε4-specific associations of VEGF gene family expression with cognitive aging and Alzheimer’s disease. Neurobiol. Aging.

[32] Tamagno E., Bardini P., Obbili A., Vitali A., Borghi R., Zaccheo D., Pronzato M.A., Danni O., Smith M.A., Perry G. (2002). Oxidative stress increases expression and activity of BACE in NT2 neurons. Neurobiol. Dis..

[33] Gonzalez-Velasquez F.J., Moss M.A. (2008). Soluble aggregates of the amyloid-beta protein activate endothelial monolayers for adhesion and subsequent transmigration of monocyte cells. J. Neurochem..

[34] Rhodin J.A., Thomas T. (2001). A vascular connection to Alzheimer’s disease. Microcirculation.

[35] Abubaker A.A., Vara D., Visconte C., Eggleston I., Torti M., Canobbio I., Pula G. (2019). Amyloid Peptide beta1-42 Induces Integrin alphaIIbbeta3 Activation, Platelet Adhesion, and Thrombus Formation in a NADPH Oxidase-Dependent Manner. Oxid. Med. Cell Longev..

[36] Kucheryavykh L.Y., Davila-Rodriguez J., Rivera-Aponte D.E., Zueva L.V., Washington A.V., Sanabria P., Inyushin M.Y. (2017). Platelets are responsible for the accumulation of beta-amyloid in blood clots inside and around blood vessels in mouse brain after thrombosis. Brain Res. Bull..

[37] Berlit P., Keyvani K., Kramer M., Weber R. (2015). [Cerebral amyloid angiopathy and dementia]. Nervenarzt.

[38] Jarre A., Gowert N.S., Donner L., Munzer P., Klier M., Borst O., Schaller M., Lang F., Korth C., Elvers M. (2014). Pre-activated blood platelets and a pro-thrombotic phenotype in APP23 mice modeling Alzheimer’s disease. Cell. Signal..

[39] Visconte C., Canino J., Guidetti G.F., Zara M., Seppi C., Abubaker A.A., Pula G., Torti M., Canobbio I. (2018). Amyloid precursor protein is required for in vitro platelet adhesion to amyloid peptides and potentiation of thrombus formation. Cell. Signal..

[40] Puig K.L., Combs C.K. (2013). Expression and function of APP and its metabolites outside the central nervous system. Exp. Gerontol..

[41] Greco S., Zaccagnini G., Fuschi P., Voellenkle C., Carrara M., Sadeghi I., Bearzi C., Maimone B., Castelvecchio S., Stellos K. (2017). Increased BACE1-AS long noncoding RNA and beta-amyloid levels in heart failure. Cardiovasc. Res..

[42] Kramer L.M., Brettschneider J., Lennerz J.K., Walcher D., Fang L., Rosenbohm A., Balakrishnan K., Benckendorff J., Moller P., Just S. (2018). Amyloid precursor protein-fragments-containing inclusions in cardiomyocytes with basophilic degeneration and its association with cerebral amyloid angiopathy and myocardial fibrosis. Sci. Rep..

[43] SenBanerjee S., Lin Z., Atkins G.B., Greif D.M., Rao R.M., Kumar A., Feinberg M.W., Chen Z., Simon D.I., Luscinskas F.W. (2004). KLF2 Is a novel transcriptional regulator of endothelial proinflammatory activation. J. Exp. Med..

[44] Papafaklis M.I., Takahashi S., Antoniadis A.P., Coskun A.U., Tsuda M., Mizuno S., Andreou I., Nakamura S., Makita Y., Hirohata A. (2015). Effect of the local hemodynamic environment on the de novo development and progression of eccentric coronary atherosclerosis in humans: Insights from PREDICTION. Atherosclerosis.

[45] Feng S., Bowden N., Fragiadaki M., Souilhol C., Hsiao S., Mahmoud M., Allen S., Pirri D., Ayllon B.T., Akhtar S. (2017). Mechanical Activation of Hypoxia-Inducible Factor 1alpha Drives Endothelial Dysfunction at Atheroprone Sites. Arter. Thromb. Vasc. Biol..

[46] Thilo F., Vorderwulbecke B.J., Marki A., Krueger K., Liu Y., Baumunk D., Zakrzewicz A., Tepel M. (2012). Pulsatile atheroprone shear stress affects the expression of transient receptor potential channels in human endothelial cells. Hypertension.

[47] Salminen A., Kauppinen A., Kaarniranta K. (2017). Hypoxia/ischemia activate processing of Amyloid Precursor Protein: Impact of vascular dysfunction in the pathogenesis of Alzheimer’s disease. J. Neurochem..

[48] Lange-Dohna C., Zeitschel U., Gaunitz F., Perez-Polo J.R., Bigl V., Rossner S. (2003). Cloning and expression of the rat BACE1 promoter. J. Neurosci. Res..

[49] Kwak M., Hong C., Myeong J., Park E.Y.J., Jeon J.H., So I. (2018). Galphai-mediated TRPC4 activation by polycystin-1 contributes to endothelial function via STAT1 activation. Sci. Rep..

[50] Garcia-Cardena G., Comander J., Anderson K.R., Blackman B.R., Gimbrone M.A. (2001). Biomechanical activation of vascular endothelium as a determinant of its functional phenotype. Proc. Natl. Acad. Sci. USA.

[51] Deng X., Zhang J., Liu Y., Chen L., Yu C. (2017). TNF-alpha regulates the proteolytic degradation of ST6Gal-1 and endothelial cell-cell junctions through upregulating expression of BACE1. Sci. Rep..

[52] Chappell D.C., Varner S.E., Nerem R.M., Medford R.M., Alexander R.W. (1998). Oscillatory shear stress stimulates adhesion molecule expression in cultured human endothelium. Circ. Res..

[53] Puglielli L., Friedlich A.L., Setchell K.D., Nagano S., Opazo C., Cherny R.A., Barnham K.J., Wade J.D., Melov S., Kovacs D.M. (2005). Alzheimer disease beta-amyloid activity mimics cholesterol oxidase. J. Clin. Investig..

[54] Kim J., Basak J.M., Holtzman D.M. (2009). The role of apolipoprotein E in Alzheimer’s disease. Neuron.

[55] Pellegrini L., Passer B.J., Tabaton M., Ganjei J.K., D’Adamio L. (1999). Alternative, non-secretase processing of Alzheimer’s beta-amyloid precursor protein during apoptosis by caspase-6 and -8. J. Biol. Chem..

[56] Gervais F.G., Xu D., Robertson G.S., Vaillancourt J.P., Zhu Y., Huang J., LeBlanc A., Smith D., Rigby M., Shearman M.S. (1999). Involvement of caspases in proteolytic cleavage of Alzheimer’s amyloid-beta precursor protein and amyloidogenic A beta peptide formation. Cell.

[57] Prabhakar N.R., Semenza G.L. (2012). Adaptive and maladaptive cardiorespiratory responses to continuous and intermittent hypoxia mediated by hypoxia-inducible factors 1 and 2. Physiol. Rev..

[58] Canobbio I., Catricala S., Di Pasqua L.G., Guidetti G., Consonni A., Manganaro D., Torti M. (2013). Immobilized amyloid Abeta peptides support platelet adhesion and activation. FEBS Lett..

[59] Azevedo E.P., Guimaraes-Costa A.B., Torezani G.S., Braga C.A., Palhano F.L., Kelly J.W., Saraiva E.M., Foguel D. (2012). Amyloid fibrils trigger the release of neutrophil extracellular traps (NETs), causing fibril fragmentation by NET-associated elastase. J. Biol. Chem..

[60] Tan J., Town T., Suo Z., Wu Y., Song S., Kundtz A., Kroeger J., Humphrey J., Crawford F., Mullan M. (1999). Induction of CD40 on human endothelial cells by Alzheimer’s beta-amyloid peptides. Brain Res. Bull..

[61] Sun X., He G., Qing H., Zhou W., Dobie F., Cai F., Staufenbiel M., Huang L.E., Song W. (2006). Hypoxia facilitates Alzheimer’s disease pathogenesis by up-regulating BACE1 gene expression. Proc. Natl. Acad. Sci. USA.

[62] Ge Y.W., Maloney B., Sambamurti K., Lahiri D.K. (2004). Functional characterization of the 5’ flanking region of the BACE gene: Identification of a 91 bp fragment involved in basal level of BACE promoter expression. FASEB J..

[63] Christensen M.A., Zhou W., Qing H., Lehman A., Philipsen S., Song W. (2004). Transcriptional regulation of BACE1, the beta-amyloid precursor protein beta-secretase, by Sp1. Mol. Cell Biol..

[64] Cho H.J., Kim S.K., Jin S.M., Hwang E.M., Kim Y.S., Huh K., Mook-Jung I. (2007). IFN-gamma-induced BACE1 expression is mediated by activation of JAK2 and ERK1/2 signaling pathways and direct binding of STAT1 to BACE1 promoter in astrocytes. Glia.

[65] Buggia-Prevot V., Sevalle J., Rossner S., Checler F. (2008). NFkappaB-dependent control of BACE1 promoter transactivation by Abeta42. J. Biol. Chem..

[66] Friedrich R.P., Tepper K., Ronicke R., Soom M., Westermann M., Reymann K., Kaether C., Fandrich M. (2010). Mechanism of amyloid plaque formation suggests an intracellular basis of Abeta pathogenicity. Proc. Natl. Acad. Sci. USA.

[67] Granger D.N., Kvietys P.R. (2015). Reperfusion injury and reactive oxygen species: The evolution of a concept. Redox. Biol..

[68] Siriussawakul A., Zaky A., Lang J.D. (2010). Role of nitric oxide in hepatic ischemia-reperfusion injury. World J. Gastroenterol..

[69] Khanna A., Cowled P.A., Fitridge R.A. (2005). Nitric oxide and skeletal muscle reperfusion injury: Current controversies (research review). J. Surg. Res..

[70] Kalogeris T., Baines C.P., Krenz M., Korthuis R.J. (2012). Cell biology of ischemia/reperfusion injury. Int. Rev. Cell Mol. Biol..

[71] Yin K.J., Lee J.M., Chen S.D., Xu J., Hsu C.Y. (2002). Amyloid-beta induces Smac release via AP-1/Bim activation in cerebral endothelial cells. J. Neurosci..

[72] Abe H., Semba H., Takeda N. (2017). The Roles of Hypoxia Signaling in the Pathogenesis of Cardiovascular Diseases. J. Atheroscler. Thromb..

[73] Safronova O., Morita I. (2010). Transcriptome remodeling in hypoxic inflammation. J. Dent. Res..

[74] Pluta R. (2002). Glial expression of the beta-amyloid peptide in cardiac arrest. J. Neurol. Sci..

[75] Lutz J., Thurmel K., Heemann U. (2010). Anti-inflammatory treatment strategies for ischemia/reperfusion injury in transplantation. J. Inflamm..

[76] Alasmari F., Alshammari M.A., Alasmari A.F., Alanazi W.A., Alhazzani K. (2018). Neuroinflammatory Cytokines Induce Amyloid Beta Neurotoxicity through Modulating Amyloid Precursor Protein Levels/Metabolism. Biomed. Res. Int..

[77] Itkin A., Dupres V., Dufrene Y.F., Bechinger B., Ruysschaert J.M., Raussens V. (2011). Calcium ions promote formation of amyloid beta-peptide (1-40) oligomers causally implicated in neuronal toxicity of Alzheimer’s disease. PLoS ONE.

[78] Ding Z., Liu S., Wang X., Mathur P., Dai Y., Theus S., Deng X., Fan Y., Mehta J.L. (2016). Cross-Talk Between PCSK9 and Damaged mtDNA in Vascular Smooth Muscle Cells: Role in Apoptosis. Antioxid. Redox. Signal..

[79] Ding Z., Liu S., Wang X., Deng X., Fan Y., Shahanawaz J., Shmookler Reis R.J., Varughese K.I., Sawamura T., Mehta J.L. (2015). Cross-talk between LOX-1 and PCSK9 in vascular tissues. Cardiovasc. Res..

[80] Ding Z., Liu S., Deng X., Fan Y., Wang X., Mehta J.L. (2015). Hemodynamic shear stress modulates endothelial cell autophagy: Role of LOX-1. Int. J. Cardiol..

[81] Picard C., Poirier A., Belanger S., Labonte A., Auld D., Poirier J., Group P.-A.R. (2019). Proprotein convertase subtilisin/kexin type 9 (PCSK9) in Alzheimer’s disease: A genetic and proteomic multi-cohort study. PLoS ONE.

[82] Courtemanche H., Bigot E., Pichelin M., Guyomarch B., Boutoleau-Bretonniere C., Le May C., Derkinderen P., Cariou B. (2018). PCSK9 Concentrations in Cerebrospinal Fluid Are Not Specifically Increased in Alzheimer’s Disease. J. Alzheimers. Dis..

[83] Apaijai N., Moisescu D.M., Palee S., McSweeney C.M., Saiyasit N., Maneechote C., Boonnag C., Chattipakorn N., Chattipakorn S.C. (2019). Pretreatment With PCSK9 Inhibitor Protects the Brain Against Cardiac Ischemia/Reperfusion Injury Through a Reduction of Neuronal Inflammation and Amyloid Beta Aggregation. J. Am. Heart Assoc..

[84] Storck S.E., Meister S., Nahrath J., Meissner J.N., Schubert N., Di Spiezio A., Baches S., Vandenbroucke R.E., Bouter Y., Prikulis I. (2016). Endothelial LRP1 transports amyloid-beta(1-42) across the blood-brain barrier. J. Clin. Investig..

[85] Canuel M., Sun X., Asselin M.C., Paramithiotis E., Prat A., Seidah N.G. (2013). Proprotein convertase subtilisin/kexin type 9 (PCSK9) can mediate degradation of the low density lipoprotein receptor-related protein 1 (LRP-1). PLoS ONE.

[86] Pluta R. (2000). The role of apolipoprotein E in the deposition of beta-amyloid peptide during ischemia-reperfusion brain injury. A model of early Alzheimer’s disease. Ann. N. Y. Acad. Sci..

[87] Stakos D.A., Stamatelopoulos K., Bampatsias D., Sachse M., Zormpas E., Vlachogiannis N.I., Tual-Chalot S., Stellos K. (2020). The Alzheimer’s Disease Amyloid-Beta Hypothesis in Cardiovascular Aging and Disease: JACC Focus Seminar. J. Am. Coll. Cardiol..

[88] Dai J.N., Zong Y., Zhong L.M., Li Y.M., Zhang W., Bian L.G., Ai Q.L., Liu Y.D., Sun J., Lu D. (2011). Gastrodin inhibits expression of inducible NO synthase, cyclooxygenase-2 and proinflammatory cytokines in cultured LPS-stimulated microglia via MAPK pathways. PLoS ONE.

[89] Tajima H., Kawasumi M., Chiba T., Yamada M., Yamashita K., Nawa M., Kita Y., Kouyama K., Aiso S., Matsuoka M. (2005). A humanin derivative, S14G-HN, prevents amyloid-beta-induced memory impairment in mice. J. Neurosci. Res..

